# Dublin Hospital Reports

**Published:** 1823-06-01

**Authors:** 


*The Dublin Hospital Reports and Communications in Medicine and Surgery.* Volume the Third, Octavo, pp. 500, three plates. Dublin, 1822.

The Dublin Hospital Reports continue to uphold their character, as unquestionably the first work of the kind which now issues from the periodical press. How long they may persevere in this course of zeal and talent, we shall not venture to predict, since the history of such works gives us the mortifying information that every publication of the kind has, in time, degenerated from its pristine vigour. This affords no proof, though a strong probability, that a similar fate awaits the work before us, as well as every other of the present day. But we have no business with prognostications; our duty is to exhibit the actual state and condition of the works before us.

This volume contains nineteen communications, the first of which, Dr. Cheyne's report on dysentery, we have analysed with Dr. O'Brien's work on the same subject, in a separate article. The second paper then is a—

*Report of Cases of Aneurism in the Richmond Surgical Hospital.* By Charles H. Todd, one of the Senior Surgeons to that Establishment.

The operations for aneurism, though now-a-days not rare nor timidly executed, are yet sufficiently interesting to deserve commemoration. The ligature of the external iliac, carotid, and subclavian arteries, is one of the proudest boasts of modern surgery.

In respect to the operation on the external iliac, Mr. Todd adopted the mode described by Sir Astley Cooper, in preference to that recommended by Mr. Abernethy. This preference was given after repeated trials on the dead and on the living subject, because "Sir Astley Cooper's method afforded the operator a greater facility of applying the ligature to the artery, more room being obtained by it, and with less disturbance of the peritoneum than by the other mode." When, however, it is necessary to apply a ligature to a higher part of the artery, Mr. Abernethy's method may, he thinks, be employed with advantage.

The two cases that terminated fatally exhibited not the slightest traces of peritoneal inflammation, and in both, the collateral circulation was quickly and effectually established.

"The cases of popliteal aneurism are related chiefly with a view of recommending a more general adoption, than is at present practised, of a preparatory course, previously to operation. I can scarcely doubt but that in many cases of aneurism, in which operations have failed, from mortification of the limbs succeeding, the patient might have been saved by a delay sufficient to allow some progress to be made in establishing the collateral circulation; and I feel confident that this desirable object may be promoted in most instances of recent disease, situated at a sufficient distance from the trunk, by compressing the principal artery of the limb for a few hours every day, for a period which must vary according to the circumstances of the case." P. 94.

In some instances, however, notwithstanding every effort to diminish the force of the circulation, an aneurism will increase rapidly—in which case, delay must not take place, and the operation must be resorted to before the tumour has acquired such magnitude as to endanger the soundness of the integuments. If the health also suffers from local pain or irritation, the operation cannot safely be postponed.

*Case* 1. A young woman, 22 years of age, of robust habit, and florid complexion, was admitted into the Richmond Hospital on the 26th of May, 1819, having a large oblong tumour on the superior and anterior part of the thigh, close to Poupart's ligament, beyond the plane of which, it projected considerably, and ascended so much, that the ligament was arched across the upper part of the swelling. Fluctuation was evident, although the tumour was tense; but no pulsation could be felt, and the superincumbent integuments were not dicoloured.

"On the inner side of this tumor, and extending to the left labium, a smaller tumor existed, between which and the large one there appeared to be a direct communication at their bases, although a depression of the skin divided them on the surface; this tumor was also tense, with fluctuation; but its integument was healthy. The thigh, leg, and foot were slightly œdematous; the patient complained of pain on the inside of the knee, and of some numbness of the entire limb—her pulse was perfectly regular; she had no palpitation of the heart or oppression of the chest, and her general health was in every respect unimpaired. Pressure upon the external iliac artery did not affect the size or tension of the tumors; neither were they perceptibly diminished by pressure directly applied to them. No pulsation could be discovered in the course of any of the large arteries below the tumor, although the temperature of the limb corresponded exactly with that of the other." 97.

She said that for four or five months previously, she had felt pains about the groin, at first slight, but gradually increasing. She next perceived the tumour, but denied the existence of pulsation in it at any period.

Two days after admission, (28th May) our author proceeded to make an oblique puncture into the tumour with a very small lancet, when a dark-coloured fluid blood flowed from the wound in a gentle but continued stream. When a director was introduced, the blood flowed more rapidly, and carried with it small coagula and particles of colourless fibrine. The wound was closed by adhesive plaster. On the 1st of June, the tumour, having increased in size and become more painful, so much resembled an abscess, that another puncture was made with the same result as before. On the 2d of June, the circumference of the diseased thigh, including the tumour at its most prominent part, measured 19½ inches, while that of the other limb was three inches and a half less. In consultation, it was agreed that the ligature of the iliac artery presented the only prospect of relief, and on the 3d of June, it was performed in the manner recommended by Sir Astley Cooper. On the ligature being tightened, the patient did not complain of any particular sensation in the limb, nor was there any evident alteration produced in the tumour. In the evening there was numbness, with some decrease of temperature in the limb—pulse 100, skin hot and dry. Venesection, effervescing draughts, &c. Sickness at stomach, and pulse at 120, were noted on the 4th of June. On the 7th, there was some hæmorrhage from the tumour, which was considerably diminished in size. The bleeding was easily suppressed. On the 8th, the discharge is profuse—coagula come away freely, some resembling putrid blood—others of a yellow colour, and pulpy gelatinous consistence. We cannot follow the daily detail of symptoms; but have only to state that she died, worn out with pain, irritation, fever, and discharge, on the 17th of June, about a fortnight after the operation. We shall give the dissection in the words of our author, by which our surgical readers will be enabled to judge whether this was really an aneurism, or a fungoid or hæmatode tumour. We are inclined to the latter opinion.

"The tumor was laid open to its fullest extent, and its putrid contents having been washed out, the internal surface presented a very irregular appearance; no trace of a distinct sac could be observed. The small branches of nerves, which seemed to have resisted the putrifactive effects of the contents of the tumor, were completely insulated, and extended through the cavity unaccompanied by veins or arteries. The muscles, except where they formed the walls of the cavity, were healthy, but somewhat paler than natural. Rather more than three inches of the femoral artery was wholly destroyed; the upper extremity of it was found in a contracted state, immediately below Poupart's ligament. The lower part of the artery contained a coagulum, which filled its cavity for several inches; the outer surface of this coagulum was very firm, of the colour of fibrine, and in close apposition with the internal surface of the artery; the red globules and more liquid parts of the clot were contained within this fibrous layer, which gave the coagulum a tubular appearance. The anterior part of the crural vein, immediately below the ligament, and where it lies parallel to the artery, was destroyed for more than an inch, and thus opened into the cavity of the tumor.

"The external iliac artery contained a firm clot, which extended from the point at which the ligature was applied to the orifice of the internal iliac. This clot adhered very closely to the internal coat of the artery, and when torn from it, the latter appeared vascular and villous.

"The ligature was quite detached, and had every appearance of having been so for some days." 111.

The second case presents no ambiguity. The patient was a printer, 28 years of age, who had received a blow from the handle of the press on his left groin, about six or seven months previously. Soon after this injury, a small pulsating tumour was perceived in the part, which gradually increased, and presented the following appearances on admittance into the Richmond Surgical Hospital, on the 14th November, 1820.

"His countenance is pale and sallow; his stature is low; his limbs small, and he appears to possess a weak and delicate constitution. Immediately below Poupart's ligament, on the left thigh, there is an aneurism of the femoral artery; the tumor is as large as a swan's egg; it pulsates strongly; pressure above the tumor suspends the pulsation, and the swelling almost entirely subsides; the integuments are healthy; about two inches below this, and in the line of the femoral artery, there is another tumor of great magnitude, and of a globular figure; it extends to the internal condyle of the femur; has a tense firm feel, with an obscure fluctuation; the integuments are of a dusky yellow colour, and are traversed by three or four enlarged veins." 112.

When horizontal, no pain in either tumour. There is some numbness in the leg. He was kept in the hospital till the 4th of December, during which time his general health improved by proper diet and medicines. On the above day, Mr. Todd tied the external iliac artery without any difficulty. The ligature drawn, the pulsation in the inguinal tumour ceased, and the tumour itself became flaccid. The lower tumour remained unaltered. On the 7th, we observe that there was some tenderness near the wound, the pulse being 112. Venesectio ad ℥x. The inguinal tumour has acquired a solid feel, and pressure has no effect in diminishing its bulk. The lower tumour is smaller, and much softer than before the operation. On the 8th, he was again bled, having some uneasiness in the lower part of the abdomen, with head-ache. On the 25th, the ligature came away—the wound is healing—the tumours much diminished, and free from pain—the countenance improved—good appetite—bowels regular—pulse 80. On the 28th, a profuse hæmorrhage suddenly took place *per saltum*. Pressure stopped the flow—a composing draught was exhibited—and the strictest quietude enjoined. The wound was left uncovered. December 30th, a hæmorrhage this evening to the amount of six ounces, which was suppressed, as before, by moderate pressure. His bowels are confined, and a circumscribed tumour, with pulsation, is indistinctly felt, in the abdomen, to the left side of the umbilicus. No hæmorrhage recurred after this till the 15th of January, when a considerable flow took place. It was now determined to apply a ligature to a higher part of the artery.

"While we were fixing the patient on the operation table, a profuse hæmorrhage took place, which was stopped by Mr. Cusack pressing with his finger at the bottom of the wound, until I inclosed the iliac artery in a ligature. This was effected without difficulty, an incision having been made through the abdominal parieties, in the direction of the artery, as recommended by Mr. Abernethy, so that this and the former incision somewhat resembled an inverted *T*. The patient bore the operation with great fortitude, and his progress was as favourable as could have been expected for several days. On the evening of the 21st, however, he had a very severe rigor, which continued for almost an hour; this was succeeded by an hot fit of a few minutes duration, and the paroxysm terminated in a profuse sweat, which lasted for some hours, and weakened him exceedingly." 119.

On the 22d of January, symptoms of trismus, and, ultimately, of tetanus, came on, and terminated the patient's existence in two days.

"*Dissection.* The larger tumor was found to consist of an irregular sac, with which the femoral artery communicated by a small aperture, which had the appearance of a rent in the coats of the artery anteriorly; the sac was formed of all the contiguous structures, and its internal surface was dark coloured, and very unequal; its contents were grumous blood, almost in a state of solution, and some soft coagula of lymph.

"The femoral artery, at the place of the first ligature, was open at both extremities, but the superior orifice appeared actually dilated; and we could not discover that any process had taken place here tending to the obliteration of the artery. It was a matter of surprise to those who attended the dissection that hæmorrhage had not sooner occurred, and that it did not prove immediately fatal.

"The ligature applied in the second operation was completely detached from the artery, and lay loose in the wound; here the orifices of the artery were closed, and a coagulum of more than an inch in length occupied the upper extremity." 121.

The third case, was a farmer, aged 30 years, of a florid complexion, and delicate habit, admitted the 30th of June, 1820, for aneurism in the right ham. After hard labour in ploughing, five weeks previously, a pain and swelling of the leg took place, and soon a tumour in the ham was perceived, rather larger than an egg, and pulsating strongly. The contents of the sac were quite fluid, as it could be emptied completely by pressure. The disease being so recent, the patient was confined to the horizontal posture, put on low regimen, and occasionally bled and purged. Our author next contrived an ingenious truss, the pad of which being placed in the line of the crural artery, controlled the circulation in the femoral artery, at the same time that the collateral vessels suffered little or no compression. After a trial of several weeks, the patient could not be persuaded that any advantage was gained. The tumour, however, had obviously diminished, and its contents had acquired a firm consistence. The patient complaining that his health and spirits were suffering by the confinement and low regimen, it was determined to operate. In this case, two ligatures were applied, though our author considers, that little or no advantage is thereby gained, but that, on the contrary, the second ligature may prove an additional source of irritation. He proved a very troublesome patient—much constitutional disturbance ensued—but he was ultimately discharged cured, the knee-joint being still very stiff, but the tumour much diminished. He was afterwards seen in good health and the tumour quite gone.

The fourth case was very similar to the foregoing. Preparatory treatment was employed, and the operation succeeded perfectly. The fifth case was an aneurismal tumour on the posterior part of the right fore-arm, for which Mr. Todd tied the brachial artery, at about two inches above the internal condyle of the humerus, when the pulsation in the tumour, and in the radial and ulnar arteries, immediately ceased, the pain, also, greatly subsiding. This patient perfectly recovered.

In a subsequent part of the volume, Mr. Todd has related a case of unusually large aneurism of the right axillary artery, in which the subclavian was tied. This case will be found detailed in the third volume of this Review, page 402, *et seq.* and forms one of the noblest trophies of modern surgery.

The next paper in the volume of transactions under review is a—

II.

*Report on Puerperal Fever, in answer to Queries from the General Board of Health.* By JOHN C. DOUGLAS, M.D.

DR. Douglas admits, that the present paper bears the character of "a hasty composition," and that it would admit of amplification; but for our own parts, we often prefer a hasty to a studied composition, in the relation of medical facts, as being less liable to a *tinge* in passing rapidly through the channel of the mind.

To the first Query, respecting the epidemic and recurrent character of puerperal fever, Dr. Douglas answers that, except in the lying-in hospital, he cannot say that he ever saw the disease epidemic. In the hospital he resided during the years 1809-10-11, and in that time, puerperal peritonitis "was decidedly *endemial.*" The sporadic cases of the disease met with in private practice, he thinks, may be subdued in the course of a few days, if judiciously treated. He imagines that the fatal cases of the casual fever, whether in hospital or private practice, would scarcely rate so high as one in six; "whereas, in the truly epidemic fever, hardly one half of those absolutely attacked recover." This last he considers to be a very aggravated modification of, if not a quite different disease from the sporadic fever.

To the second Query, whether he has known the term puerperal fever applied to diseases differing in their nature? our author answers—

"It is customary to apply the term puerperal fever to diseases very considerably, although not entirely different; different both with respect to their exciting cause, and as to the type of their pyrexia, although always similar in their great leading characteristic—abdominal inflammation and pain. I have for many years been of opinion, that there is not a greater difference in the type of that pyrexia, which accompanies a case of any purely inflammatory disease, as phrenitis, pleuritis, &c. and from such down through every grade of fever to the plague itself, than there is between the type of the pyrexia, attending a casual* case of puerperal fever, and of that at-

* "By the term casual case of puerperal fever, I mean such as occasionally occurs, both in hospital and private practice, when the disease is tendant on the true puerperal epidemic; and I feel myself justified in attributing the discrepancies of opinion, every where existing with regard to the nature, and the various and even opposite modes of treatment recommended by different authors for the cure of puerperal fever, to deficiency of nosological distinction." 144.

To the third Query, is it contagious when epidemic? Dr. Douglas answers in the affirmative, "but, for the most part, only to lying-in women." He believes, however, that a woman, either pregnant or while nursing, or even a very delicate female for several months after lying-in, might be susceptible of the disease. Cases occurring under these different circumstances have happened within his own knowledge. He is apprehensive that the contagion may be conveyed by persons much engaged in hospital duty, at a time when its atmosphere is heavily loaded with this peculiar effluvium.* The remaining Queries and Answers are not particularly interesting or conclusive; we shall, therefore, pass them over, in order to take some notice of the appended "Observations."

Our author proposes to divide puerperal fever into three species, *viz.*—

Synochal puerperal fever,
Gastro-bilious puerperal fever,
Epidemical or contagious puerperal fever.

"The cases that I rank under the first distinction are all those attended with pyrexia, similar to that of any other purely inflammatory disease. These are neither produced by contagion, nor are they themselves infectious. They are usually the result either of tedious or ill-managed labour, or of exposure to cold or other adventitious cause, after delivery.

"This form of the disease is to be subdued by copious blood-letting, and other sedative and evacuant remedies, viz. antimonials, purgatives, enemata, fomentations, &c. The abstraction of blood, however, should here be considered our sheet-anchor: this fluid must not only be early and copiously taken, but the operation should be repeated in proportion as the pulse or abdominal pain may afterwards indicate. The pulse, I may remark, is often in this, as in every variety of abdominal inflammation, deceitful. I therefore regard the degree of pain felt on pressure, as a preferable criterion, by which to regulate the abstraction of blood; and I cannot refrain from stating it to be my opinion, that the reason of this inestimable remedy having

---

not epidemic, and which is excited by accidental causes, either during labour, or subsequently to delivery. In these cases, I presume the local inflammation more frequently to be primary, and the pyrexia to be consequential; whereas, in the epidemical, I consider the pyrexia to be primary, and the local affection to be consequential."

* He has generally perceived by the sense of smell, the effluvia of puerperal fever, when epidemic, on entering the wards.

so frequently failed of the desired effect, and having been, by some condemned as useless or even injurious in this and other diseases, is not owing to genuine inaptiude in the remedy, but to the inefficient manner in which it is often applied by timid practitioners." 153.

Under the second species our author includes those cases wherein the disease does not so rapidly assume a decidedly inflammatory character—where the fever does not, as in the former case, commence with a bounding, incompressible pulse ; "but with a pulse frequent, hard, and concentrated ; neither are the symptoms of abdominal inflammation so early evolved." Yet such inflammation, our author observes, does exist, and progresses, when not checked, although more slowly and more obscurely than in the former species. The tongue is here loaded as in common bilious fever ; "whilst in the former it is usually white, or cleanly florid, with sometimes a glazed appearance."

"The treatment here should likewise commence with blood-letting, but in more moderate quantity ; immediately after which ten or twelve grains of calomel should be administered, and followed in a few hours by an ounce of castor oil combined with some other briskly purgative medicine. These medicines, unless given in large doses, will produce but little effect, in this complaint ; and it is of paramount importance that the mucous surface of the intestinal canal should be effectually acted upon early in the disease in all cases, and more particularly whilst the morbid action of the peritoneal covering is under a kind of panic from the blood-letting, and before that inflammatory action can re-organize its broken force. Often, by such prompt treatment, will the disease be arrested *in limine* ; should it however advance, whether in increased or subdued violence, purgative medicines, varying in kind, must be daily administered, assisted by enemata, fomentations, topical blood-letting, &c." 154.

That form which our author arranges under the third head, he considers to be "the really contagious or epidemical puerperal fever ; and although agreeing with the others in the great leading symptoms, inflammation, pain, tumefaction and tension of the abdomen, yet differing from them in many material characters."

"The sensorium here is seldom in any degree disturbed ; whereas in the others it is so, frequently, and even sometimes is excited to high delirium. The pulse here is usually from the moment of attack, soft, weak and yielding, and in quickness often exceeds 160, whereas in the first species it is full, bounding, and incompressible ; and in the second, small, hard, and concentrated, and in both moderately quick. The eye, instead of being suffused with a reddish or yellow tint, as in the others, is here generally pellucid, with dilated pupil. The countenance, instead of being being flushed as in the others, is here pale and shrunk, with an indescribable expression of anxiety ; an expression altogether so peculiar, that the disease could on many occasions, be pronounced or inferred from the countenance alone. The surface of the body, instead of being as in the others, dry and of high pyrexial heat, is here usually soft and clammy, and of heat not above the natural temperature, and not only is the skin cool with clammy exudation, but the muscles to the impression of the finger, feel soft and flaccid as if deprived of their *vis insita*, by the influence of the contagion. Indeed there is such prostration of muscular strength, and depression of vital principle, from the very onset of the attack, that I must suppose the contagion to act upon the human frame, probably through the medium of the nervous system, in a manner analogous to that of the contagion of the plague; and perhaps the African plague does not commit greater havoc among an equal number of infected persons than puerperal fever in this country; nor is puerperal fever less quickly fatal than the plague itself." 156.

It appears to us that this form of fever does not agree with the general descriptions of epidemic fevers in other times and places, as given by various authors. We must therefore suppose, what indeed is very probable, that the epidemic of Dublin differed in character from the epidemic of Edinburgh and other places, thus requiring a modification of treatment. It will not fail to strike the notice of our readers that the above extract conveys a tolerable idea of what has been described in this country as the *congestive* form of puerperal fever—a form by no means common in the epidemics of this country and Scotland. We suppose the climate and habits of the Irish must be the *principal* modifying causes. The mode of treatment recommended by Dr. Douglas we shall give in his own words.

"Without pretending to detail any of the various modes of treatment, which I may have seen pursued either successfully or otherwise, I would here recommend the practitioner to commence by administering ten grains of calomel, combined with two grains of powdered opium, in the form of bolus, or of pills; likewise, as early as possible, a briskly purgative enema. After the operation of the enema a number of leeches, from two to four dozen, according to circumstances, should be applied to the abdomen, and the abdomen should afterwards be stuped with flannel clothes, wrung from warm water; and not only at this period, but frequently through the whole course of the disease, should such fomentations be used. Three or four hours having elapsed from the time of administering the calomel and opium, three drachms of pure oil of turpentine, with three drachms of syrup and six drachms of water, in the form of a draught, should be swallowed; and, after the lapse of another hour, this is to be followed by an ounce of castor oil, or some other briskly purgative medicine. In some instances, the oil of turpentine and castor oil may be combined in one draught; but I generally prefer giving the turpentine as here recommended. Some of these remedies, occasionally assisted by others suitable to the peculiarities of the case, are to be repeated as circumstances may indicate; but I would not be disposed to repeat the internal use of turpentine oftener than twice, in any case whatever. In several cases, particularly where the debility is very considerable, the blood-letting may be altogether omitted, and in these cases a flannel-cloth, sopped in oil of turpentine, should be applied to the abdomen, and allowed to remain on for the space of about fifteen minutes. This external application of turpentine, without either its internal use, or the aid of blood-letting, I have frequently experienced to be entirely efficacious in curing puerperal attacks; and although I have hitherto omitted to speak of turpentine for the cure of the other varieties of this disease, yet I would not feel as if I were doing justice to the community, if I did not distinctly state that I consider it, *when judiciously administered*, more generally suitable, and more effectually remedial, than any other medicine yet proposed. I can safely aver I have seen women recover, apparently by its influence, from almost hopeless conditions; *certainly* after every hope of recovery, under ordinary treatment, had been relinquished." 157.

Our author does not pretend to account for the *modus operandi* of the oil of turpentine in these cases. He thinks that where it acts as a purgative, there are rational and philosophic grounds for concluding that the medicine excites powerfully the whole mucous membrane of the intestines, and thus derives the morbid irritation from the peritoneal tunic to a secreting surface, where it is carried off by the increase of secretion itself. But, on the other hand, he has known the turpentine in several instances, even by its external application, relieve the most urgent symptoms of this formidable disease in the course of fifteen or twenty minutes, without producing any sensible evacuation. We do not think that this fact is at all inexplicable on the abovementioned principle of counter-irritation. If the irritation can be transferred to the external surface of the body, it is still better than to the internal. Our author regrets the discrepancies of opinion respecting this medicine, which are scattered in our periodical works. His own conviction is, that the epidemic puerperal fever of lying-in hospitals, is "neither more nor less than a malignant fever of a typhoid character, accompanied with an *erysipelatous inflammation* of the peritoneal covering of the stomach, intestines, and other abdominal viscera." We have heard this remark made by some very old practitioners in this metropolis; but on pressing them for particulars, we found they could shew us no satisfactory proofs of this erysipelatous inflammation as differing from the usual phlogosis of the serous membranes in general. Their dissections exhibited reddening of the membrane—adhesions,—and sero-purulent effusion. If

Dr. Douglas's dissections disclosed any anatomical proofs of an inflammation differing in these respects, he certainly should have stated them ; and till then, we shall beg leave to withhold our assent from this assumed division into phlegmonous and erysipelatous inflammation of the peritoneum. By this we do not mean to assert that all cases of puerperal peritonitis will bear vigorous depletion ; we have all along expressed our conviction that there are epidemics, where the accompanying fever is of that kind that will not bear depletion as in the sporadic attacks. We agree with our author in the concluding passage of his paper.

"It must, however, be distinctly understood, that I here allude to the type of the pyrexia of epidemic fevers in Lying-in Hospitals. For I am led to believe, when puerperal fever happens to be epidemic at large, in towns or districts, that its pyrexia is rather of the synochal character, and requires for its subjugation the bold and decisive depletions, recommended in Dr. Armstrong's invaluable treatise on this subject." 160.

Upon the whole, we think our professional brethren will be disposed to return thanks to Dr. Douglas for pourtraying the results of his experience on the important topics discussed in the above paper.

The next paper in this volume is a—

III.

*Medical Report, containing an Inquiry into the Causes and Character of the Diseases of the Lower Orders of Dublin.* By T. C. Speer, M.D. M.R.I.A. late Physician to the Dublin General Dispensary.

Man is a component part of the earth under his feet and of the elements around him—to which is added a vital principle or organization that leaves him in a condition to be influenced by, but not entirely subject to, the laws of the circumambient unorganized matter. We have no doubt, indeed, that national character, as well as form and other material properties, depends as much on climate and physical circumstances, as on government, laws, religion, and education. Philosophers have attributed too little to the former, and too much to the latter agents. Our author thinks, and not without reason, that Ireland furnishes an illustration of this proposition.

"Irish character, like Irish climate, is full of peculiarities, and without being fanciful, I think we may trace a congeniality between them ; like the climate, it abounds in vicissitudes, varieties, and extremes ; between the bright and dark, little medium is observed. It is to the lower orders, of course, that this observation chiefly applies ; with them civilization seems chiefly made up of two of the materials above mentioned,* viz. education and amenability to the laws. Now these are the points in which our lower orders, as compared with those of our neighbours, seem particularly backward; two, therefore, out of the four moral causes being more or less withdrawn, the influence of natural causes, such as climate, must, with us, be infinitely greater." 164.

While remarking on the diseases of the lower classes, our author makes some keen reflections on the advantages and disadvantages (to the medical practitioner) of Dispensary practice. As this practice is now pretty generally monopolized by our junior brethren, we shall give Dr. Speers' sentiments on the subject, in hopes that they may profit by his observations.

"The practice of a general Dispensary is perhaps the most complete introduction to the diseases and indeed to the distresses, habits, and character of the lower orders of a city. Sorrows and sufferings are here unveiled, which shame will hide from the public eye; here we shall see how the chain of poverty has its various links, and the cup of bitterness its various dregs; here we become associated with disease in all its varied and complicated shapes, and here we are most promptly and powerfully called on to combat it with the rules of our art.

"Dispensary practice has its advantages and disadvantages pretty equally mixed; it creates promptitude; it affords a wide range of insight into local peculiarities; it opens an immense and diversified page, not only in the book of medicine, but of mankind; it breaks away the fancies of the closet, and the bondage of the schools, and gives confidence and courage at the bed-side of a patient. On the other hand it has its disadvantages; it creates a coarseness of practice; we know we are dealing with raw and uncertain materials, and we find the most common plans of treatment often answer the best; by these habits our thinking and theorizing powers are weakened and hurt; we cannot gain very much as to the effects of our medicines, because we know these effects may be and are counteracted by improper diet and regimen. We cannot, as in Hospital practice, restrict our patients to certain rules and laws, nor confide them to nurses or even friends who can be relied upon. We reason, therefore, much less on the *modus operandi* of our medicines, and thus an unfair spirit of distrust and empiricism may be generated." 167.

There is another inconvenience attending dispensary practice among our younger brethren, far greater than those delineated by Dr. Speer. It is this:—diseases, when caused or accompanied by want, the depressing passions, filth, and unwholesome food, are greatly modified thereby, and vary much from diseases of a similar kind among the higher

---

* Government, laws, religion, and education.

classes of society, where, in fact, the circumstances are diametrically opposite. The young practitioner, therefore, who has not a proper balance of practice among the different classes, must necessarily form erroneous opinions and contract partial views of diseases, upon several occasions. Still the Dispensary is the very best school *he* can have; and time will correct the imperfections from which no institution is free.

The causes which lead to the formation of disease in the lower orders of the Dublin population, our author classes under the heads of climate, poverty, population, and national character.

1. *Climate.* The thermometric range is not near so great in Dublin as in England or Paris. The summer heat seldom exceeds 75°, and in winter the thermometer is rarely below 25°. But *within* the above range, the vicissitudes are more sudden in Dublin than in the other capitals. "Even in the twenty-four hours, variations will amount to 20, 25 or 30 degrees, so that we have often seasons of the day as well as seasons of the year." But though very fickle in its temperature, the atmosphere of Dublin is pretty regular in its currents of air. The westerly and southwesterly are the great trade or reigning winds, and are to all the others put together as three to one. Next to variability of temperature, humidity seems the most characteristic feature in the Irish climate, not only as indicated by the rain guage but also by the hygrometer.

"Such seem to be the chief peculiarities of our climate, and we may, I believe, as compared with that of England, say, in general, that it is more free from extremes in temperature, but more variable, humid, and cloudy; that our seasons are less distinctly divided, our springs more cold and severe, our summers more wet and temperate, our autumns more alike each other, and our winters much milder. Speaking without comparison, it appears from various and numerous observations, that spring is our severest season, summer our wettest, autumn our driest and warmest, and that our winters are, except towards the close, generally mild and open." 175.

It is in the order of phlegmasiæ that we must chiefly look for the effects of the climate above described. The sanguineous system is the one principally affected—and inflammation characterises the diseases. These sudden changes of temperature acting on an unprotected, ill-fed, ill-clad population, produce more or less sudden determinations to particular organs, and proportionally derange the balance of the circulation.

" How far humidity, unacccompanied with severe cold or heat, contributes to the production of disease or health, is as yet a point not clearly settled; the latter would with us seem rather to be the case. Dr. Rutty thought our moist seasons the healthiest, at least much freer from epidemics, and his various observations go decidedly to confirm this opinion; this has also been the opinion of other observers since his time, who think that, unless with great variations of temperature, severe cold, and easterly winds, our humid seasons are in general our healthiest. A single proof of this seems to have been furnished in the year 1816, which was remarkably healthy and remarkably wet. Dr. Franklin, and Dr. Percival of Manchester, conceived that moist seasons are healthier than dry ones, ceteris paribus; and Sir John Pringle seems to have been of a similar opinion. It is only therefore in its combination with extremes and varieties of temperature, &c. that we can consider humidity in its promotion of disease, and even here we know not how far to go." 177.

Spring is the grand season in Ireland for pulmonic affections. But notwithstanding the great humidity and changeableness of the sister climate, scrofula, at least in its usual forms of development, is a rare disease comparatively with England and Scotland—and even pulmonary phthisis is far less common in Ireland than in these climates, notwithstanding the ravages of pulmonic inflammation in the former island. This fact would strengthen the doctrine of Dr. Baron and some others that the development of tubercles in the lungs is not strictly an inflammatory process, though the said development does undoubtedly occasion inflammatory action in the neighbouring parenchymatous structure of the lungs.

2. *Poverty*. This is a fertile cause of disease, especially in Ireland. Potatoes, salt fish, and tea are the principal articles of Irish diet among the lower classes. Flesh meat can seldom be procured, and of fish, herrings are the favourite species—probably from their cheapness and stimulating or savoury quality.

" It is impossible not to notice the preference which the lower orders here, compared with those of England, have to *stimulating* or flavorous, rather than *nourishing* food, and indeed their carelessness about the latter except potatoes. This may be partly explained from their national character, as will hereafter be mentioned. In the diet of the lower orders in England, nourishment is the grand object, and the rules of their diet are conducted with a system and an arrangement completely unkown here. With us what excites is the chief consideration, and as to regularity in meals the poor are very indifferent about it. With the former the *stomach* is the presiding organ; it seems to hold dominion over all the others, and to be a complete tyrant. With us the *nerves* appear to hold this place, and to these a great deal is sacrificed. It is impossible not to notice, with the lower orders in England, when ill, that their great and first complaint is 'they can't eat;' here the general complaint is, that 'they have a fluttering or oppression about the heart." 181.

It is curious that notwithstanding this poverty of food and carelessness of the Irish, the lower orders are capable of labour and fatigue equal, if not superior to their brethren of England or Scotland—in short, Nature seems to have gifted them with hardy and vigorous constitutions.

"Under the head of diet, *Tea* seems to hold the highest rank with our poor; unlike its more dangerous rival, whiskey, its draughts, though impoverishing, are not delirious; if it drowns sorrow, it does not drown sense; if it gilds the gloom of poverty, is not the delusion a blessing? It seems, indeed, the general panacea, always affording comfort, calmness and consolation; constituting not only the leading article of breakfast and supper, but often of dinner, and over its placid inspirations their happiest hours seem to be passed." 183.

Unfortunately the effects of the other favourite beverage (whiskey) are of a different complexion. The great estimation, our author thinks, in which fermented liqnors are held by all northern nations, is a sure proof of their necessity and value. Among the inhabitants of Hyperborean lands there is a perpetual struggle, Dr. S. observes, between the laws of life *within*, and the laws of nature *without;* and whatever gives a preponderance to the *former* will be eagerly sought after. As we approach the Pole we observe the propensity to spirituous liquors increase. Here life is at a low ebb, artificial excitement becomes indispensible, and the means of procuring it are among the chief objects of the people.

"That the *use* of this fluid with us is attended with great advantages is unquestionable, but that its *abuse* has completely thrown these advantages into discredit, is equally so. Indeed in the entire mass of misery of our poor, whiskey is thought to form the principal remedy; they conceive it a cure for all complaints, and all weathers; in warm weather it allays their thirst; when cold, it heats them; when wet, it dries them; in sorrow they fly to it as a charm and a blessing, and in its intoxicating draughts their misery is forgotten. The bad effects, however, resulting from this fluid with us are not, I think, to be ascribed to the quantity consumed. I believe the lower orders in Manchester, Edinburgh, Glasgow, and some of the large manufacturing towns in Great Britain, consume a greater quantity; but there is a striking difference in the mode of consumption between the two—they do not take it on empty stomachs like our people; they eat much more solid nourishing food; thus the effects of the whiskey are less directed to the coats and nerves of the stomach, or to the brain, and therefore intoxication does not exhibit itself so frequently. However, the great proportion of public houses in Dublin compared with others, is a clear proof of the immense consumption of whiskey, and until such is reduced, disease and distress must stalk abroad through our streets." 184.

Our author has observed dropsical affections to constitute a most predominant feature among the diseases of the lower Irish—especially among females. This might be expected from the general indulgence in weak, watery and intoxicating fluids, indolence, poverty, &c. Their attachment to salt and salt food is constant. Salt herrings and potatoes is the standing diet.

Our author has found constipation of the bowels a remarkable and almost universal phenomenon among the class in question, whether in sickness or health, and consequently he has found no remedy so directly and almost invariably useful as purgatives. He believes that Dr. Hamilton has not in the least exaggerated their powers. Next to the elementary canal the skin seems to be principal sufferer from the effects of salt food. Cutaneous affections and obstructed perspiration are very prevalent. From the diet and drink we need not wonder at finding a large proportion of nervous affections, as low nervous fevers, dyspepsia, hypochondriasis, asthma, &c. But the liver and stomach bear the chief onus of disease. Hepatic derangements are far more prevalent among the inhabitants of Dublin than of London or Edinburgh, owing, no doubt, to the ingurgitation of ardent spirits on an empty stomach. "The dissections at the various hospitals, says our author, exhibit greater derangement of this organ (the liver) than almost any other." He has seen it assume various forms of disordered structure, and he has always observed it to be the most prolific parent of disease. "Inflammations, indurations, adhesions, and turbercles are the general appearances it presents, and I believe it is rarely free from some one of these." In the stomach are found inflammation, thickening of the coats, induration, scirrhus, &c.

3. *Population.* On the 30th of October, 1821, a census of the population of Dublin was completed, and found to be 238,201 souls. This exuberant population, combined with the decline of trade and manufactures, the early and imprudent marriages of the Irish, &c. furnishes an abundant source of disease among the inferior classes. The effects of a dense population are particularly conspicuous during epidemics. In the crowded quarters of the city the atmosphere, both in and out of doors, becomes highly vitiated, ventilation is suppressed, the windows are hermetically sealed, and crowds of human beings are constantly exchanging the exhalations of each others' lungs, and thus diffusing contagion in every shape and direction. The last subject touched upon by our author is—

4. *National Character.* Even this is by no means unconnected with health and disease. The following passage exhibits the bright or moral side of the Irish character among the lower orders of society.

" *Passion* seems to be the grand and presiding feature in the national character of these people: from this all its peculiarities emanate, and whatever it has of faults or beauties are here to be found; it is their constant guide, and regulates most of their actions. Their principle of *thinking* being so subordinate to that of *feeling*, and their principle of *feeling* being subject to such rapid changes and vicissitudes, we see about them that freedom, lightness, and carelessness, which makes them happy even in their miseries, because unmindful of them. Sorrow cannot rest upon them long at a time; their constitution is elastic, and soon shakes it off. There is a strong love of life about them, and a love of each other; and although distress and sufferings press in every shape around them, yet this love seems proportionally to increase in the strength of its instinct, and to cling more closely to them: that tedium vitæ, often so conspicuous among our neighbours, is here but rarely observed; suicide is almost unknown, and let their sufferings and privations be ever so crowded on each other, they rise superior to them, or bow with resignation. Universally good natured, careless, warm in heart, hospitable, grateful, and fond of each other; full of words, exaggerations and professions, they ever look at the bright side of life; hope is their companion, and seldom deserts them." 195.

But from this character, amiable as it is in many respects, spring great sources of suffering and degradation. They all hold the doctrine of fatalism, believing that their own exertions are of very little use, and that whatever happens is from inevitable destiny, to which they bow with placid resignation. Hence the total neglect and abandonment which marks every thing about them. Indolent, prodigal, and careless, they look not beyond the wants of to-day, contented if they can satisfy the common cravings of nature. Indolence and dirt, the result of this character of the lower Irish, are the principal agents in the production of their diseases.

" Under the head of want of cleanliness, with reference to individual health, we have chiefly to regard the condition of the skin. This acquires a complete coating, its exhalents are obstructed, perspiration is prevented and vitiated, and thus one great outlet for the discharge of impurities is closed; the proper healthy balance of circulation is destroyed, the internal organs are oppressed, and the whole machine deranged; thus we cannot wonder that cutaneous affections should form so large a proportion of disease with the Irish poor.'' 198.

Under these circumstances we cannot wonder at the rapid diffusion of contagion when once introduced or generated among such a dense population. Within doors every article of furniture and wearing apparel is disfigured with filth—every spot encrusted with its layers; and the foulest odours every where abound. Out of doors, especially in warm seasons, the church-yards, slaughter-houses, and masses of filth and offal in the narrow streets and lanes contribute no less to the propagation of contagion. In some of his visits among the narrow and crowded streets, Dr. Speer has been obliged to wade through masses of filth enough to sicken the stoutest heart—masses that must have remained there for months, perhaps for years generating the most putrid effluvia! This far exceeds any thing we see in St. Giles's! and we hope the police of Dublin will soon remove such a disgrace and danger from the sister metropolis.

The study of medical topography, and of the public sources of health and disease, is a very important and useful study; and it was with the view of exciting our junior brethren to it that we have entered so largely into the paper just now concluded. Dr. Speer is entitled to the thanks of the profession, and of his fellow citizens in particular, for pointing out so many public and private nuisances, many of which are remediable or removable by proper interference.

### IV.

### *Fatal Consequences resulting from slight Wounds received in Dissection.* By A. Colles, M.D.

A year never passes, now-a-days, without proving fatal to several of our brethren in the above way. This used to be a comparatively rare occurrence, as dissection was pretty closely confined to the dissecting room; but now there is scarcely a general practitioner who is not in the habit of making pathological researches whenever opportunity occurs, which is often the case, owing to the decrease of prejudice among all classes of society.

It is somewhat curious that, of the three histories given by Dr. Colles, under the head of "fatal consequences," &c. two terminated favourably—which reminds us of the Hibernian's propensity to cry out that he is "*murdered*," when he is only knocked down. It must be confessed, however, that of the two gentlemen who recovered, one was almost in the grave, and the other appears to have had a very narrow escape.

The operation of the septic principle received during dissection, varies very much in different individuals, so that we do not think any thing like a history of the complaint can be given. In the two worst cases here narrated, there was considerable resemblance, not only to one another, but to the case of the late Dr. Pett, of Hackney. We shall first give a sketch of the cases themselves.

*Case* 1. Mr. Hutchinson, one of Dr. Colles' pupils, received a slight scratch while opening the body of a man who had died of cynanche laryngea. The cellular membrane on the external surface of the larynx and pharynx of the deceased contained that amber-coloured fluid resembling melted jelly, which is so often met with in such as have been carried off by this disease. The scratch on the thumb was very slight, yet the gentleman was that evening drowsy, and awoke next morning with head-ache, sickness of stomach, and acute pain in the shoulder and axilla of that side. On the third morning, the pain had increased to an extraordinary degree, and was still confined to the shoulder-joint, but without any discolouration of the integuments. There were no inflamed lymphatics on the arm, nor enlargement of the axillary glands. Yet he suffered exquisite pains about the clavicle and axilla, on the slightest pressure being used. The scratch on the finger at this time was quite free from inflammation, but the cuticle was raised in a small vesicle, partially filled with a white milky fluid. In this state he continued for three or four days, in agonizing pain, violent fever, and great mental despondency. Fomentations, leeches, opiates, afforded no relief. At length, some mitigation of the pain took place, and a diffused erysipelatose redness spread along the thorax and abdomen, down near to the ilium, and ultimately to the trochanter. The skin had a doughy feel, and retained the impression of the finger. The fever continued unabated, and the strength was nearly exhausted, being only supported by large quantities of wine. Incisions were made over the fourth and fifth ribs, with the hope of finding some collection in the cellular membrane, but none was discovered. In a few days more, the inflammation ceased to extend, the cuticle began to desquamate, and the pain to diminish. At the end of three weeks from the accident, the pain had nearly subsided, and his general health was improving; but, in a day or two, pain and swelling appeared in the arm, and soon became almost insupportable. Some matter formed, and was discharged by an incision. The inflammation, also, re-appeared in the side, and ended in a small abscess. After this, the patient rapidly recovered, and went into the country.

"From the commencement of the disease his spirits were sunk; he frequently raved; his pulse throughout was never less than 120, and sometimes it was 130. His stomach was, at the commencement, very irritable, and got better; but it again became alarmingly irritable about the beginning of January. This might have been partly caused by the large quantity of opium he used, for he could not procure any relief, except by a large dose, amounting to a drachm, or four scruples of the tincture daily." 207.

*Case* 2. The second case was that of Mr. Dease, the late lamented and estimable Professor of Anatomy and Surgery to the Royal College of Surgeons of Dublin. On Saturday, the 13th of February, 1819, he lectured on the cervical nerves, the subject being a female, not more than 48 hours dead. On Sunday morning early, he awoke with a violent shivering and sickness at stomach, which lasted two hours. At eleven o'clock, he earnestly besought Dr. Colles to bleed him for a pain in his left shoulder, which was most excruciating. Dr. C. declined bleeding him till three o'clock, when urged again, he took away nearly twenty ounces of blood from his arm by a large orifice. He experienced partial relief at the beginning of the bleeding; but at nine, there was no essential mitigation of his sufferings. The fever was as high as ever. The blood was neither buffed nor cupped. Dr. C. now observed a slight fulness above the clavicle, and on pressing it with his thumb, the pain became exquisite. On Monday the 15th at eight o'clock in the morning, Dr. Colles visited Mr. Dease, and found that he had had a bad night, owing to the pain in the shoulder, on which he had now nearly one hundred leeches. In a consultation, some aperient medicine was ordered, which operated but little, and did not reduce a fulness of the abdomen, which was now conspicuous. 16th of February, the bowels had been more satisfactorily relieved, but, with little diminution of the general symptoms. He was advised to continue the use of purgatives. In the evening of this day a colourless swelling was discovered on the side of the thorax, and this, for the first time, led to the suspicion of a dissection wound, of which Mr. Dease had no recollection. Dr. Colles discoved on the dorsum of the thumb, the mark of a slight scratch which formed the diameter of a vesicle half filled with a milky fluid. The pain in the shoulder was now easier, and he could better bear pressure about the clavicle. Every evening, about six o'clock, he had an exacerbation of restlessness and depression of spirits. 17th. A bolus of calomel, with a liquid purgative draught. 18th. The medicine had operated well, and he slept four hours after taking the draught, awaking cheerful and refreshed. To have an anodyne draught at bed time. 19th. Passed a bad night, and has some delirium this morning—face has a yellow tinge—countenance sharp—pulse small. The incision where he had been bled, was inflamed in the ordinary way, and there was a small vesicle on the fore-arm, two inches below the incision, containing a milky fluid. 20th. His manner was quick this morning, and bordering on delirium—pulse 126 and smaller—the entire side, from the axilla to the hip, swelled, and studded with small elevations, appearing like vesicles, but feeling hard to the touch. There was a strong erysipelatous blush occupying a small portion in the middle of the swelled side—tongue white; abdomen full. 21st. There appeared to be an abscess, but without distinct fluctuation, near the axilla; and there was a tumour on the anterior part of the right arm, which they agreed to puncture, though the patient was fast approaching to dissolution. A small quantity of serous fluid escaped, but did not reduce the swelling. He died at ten o'clock that night. There was no dissection of the body—nor indeed could it have been of any use.

The third case is only an outline. Mr. Egan had employed himself in dissecting the body on which Mr. Dease had lectured, and on the same day, viz. Saturday, 13th of February. On Sunday evening he had a rigor followed by a febrile paroxysm. On Monday, though not well, he went into company. On Tuesday he had a hoarseness, and inflammation attacked the thumb, with pain and erysipelatous redness around it, the pains passing up along the back of the fore-arm, attended with a smart degree of fever. On Friday he complained of tenderness under the border of the pectoral muscle, where an enlarged gland could be felt. His chest appeared to be seriously affected. On Sunday, the 28th of February, Dr. Colles saw him—fever running very high—pulmonary affection and distress very great. The abscess near the axilla was now opened and discharged some purulent matter, the cavity proving to be extensive, passing across the pectoral to the latissimus dorsi muscle. He gradually recovered.*

* We copy the following account of Dr. Pett's case from our respected cotemporary, the Medical and Physical journal for February of the present year, as communicated by Mr. Travers.

" At eight o'clock on the morning of Saturday, the 28th of December, Dr. Pett assisted a medical friend in examining the body of a lady who died of peritoneal inflammation after child-birth, on the Thursday preceding. At nine o'clock the same evening, being in his usual health and spirits, and while engaged at cards, he complained of an uneasiness in the middle finger of his right hand. On minute examination, a slight

Dr. Colles has made some reflections on these cases, which he thinks present us "with the character of a disease both formidable and new."

"The severe pain felt on the point of the shoulder; the peculiar colourless swelling, or fulness above the clavicle, within a few hours after the infliction of the wound; the wound itself at this time free from inflammation or pain; no red streaks, no pain, nor even tenderness on pressure through the whole course of the limb. In short, no trace at the time of any morbid connexion between the wound

---

superficial wound was discovered. It was then touched with lunar caustic, and some time afterwards with a drop of strong sulphuric acid, neither of which applications was felt. On going to rest, he again applied the lunar caustic to the wound for the space of half a minute, which application was followed by pain, soon increasing to intensity; and, an hour after he had been in bed, he was attacked by a severe rigor, which continued more or less for three hours. The pain spread from the finger along the arm to the axilla, and was so agonizing as to lead him to observe, that he had never before known what pain was. The night he decribed as one of dreadful suffering, without intermission. In the morning, the expression of his countenance, and the altered manner of the man, struck his medical friend with instant alarm. The finger was white, and without sensation.

"At twelve o'clock on Sunday, an icision was carried through the wound to the bone, which was not felt by Dr. Pett. In the course of this day, the arm became swollen, and the superficial absorbents conspicuous from inflammation. The pain affecting the arm extended to the axilla and pectoral region. The finger in a few hours became discoloured and gangrenous as far as the second joint, where suppuration of the soft parts afterwards took place. The high excitement of the nervous system, marked by a flushed countenance, a ferrety eye, vigilance, great anxiety, short and quick respiration, rapid and voluble speech, and unnaturally irritable manner, were accompanied by a very moderate acceleration of the pulse, which soon became intermittent, and then irregular.

"On the morning of Tuesday, the arm had recovered its natural appearance; it was neither swollen nor painful, nor were any absorbent lines visible. A considerable effusion had taken place in the cellular substance of the axilla, and over the pectoral muscle: it was marked by an erythematous blush, was painful, and crepitated on pressure as in emphysema. The symptoms varied but little; but increased difficulty and hurry of respiration, and increased feebleness, were marked at each visit. He expressed a sense of confusion; but there was no further evidence of disturbed intellect than the deviation before adverted to from his natural characteristic calmness. The fulness at the axillary edge of the pectoral muscle being sensibly increased on the Wednesday morning, a lancet was pushed deeply into it, but only a bloody serum issued.

"On the Wednesday evening, about six o'clock, he died, having survived the injury about 105 hours. The body was inspected on Friday, and no recent morbid appearance whatever was discovered in the chest or abdomen. The heart was unusually large, and its substance flabby. The internal structure of the liver had undergone considerable chronic degeneration. The head was not examined." 177.

and the seat of the pain, were the most remarkable features of this disease." 217.

Another remarkable phenomenon was the peculiar appearance of the pustules, (unlike to any he had ever witnessed) particularly the pustule on the fore-arm of Mr. Dease, which could not have been from the wound made by the lancet, as this pustule was *below* the incision, the intervening skin between it and the pustule being natural. The resemblance of these elevations to vesications, while to the touch they felt solid, was another peculiarity. Our author cannot agree that the swelling on the side of the chest, in these cases, was of an erysipelatous nature, as the redness was of a bright healthy colour, and did not occupy more than a sixth part of the swelling.

Dr. C. observes that, however we may dispose of the disease, in a nosological way, it is obvious that neither habits of dissection the most lengthened, nor a state of dead body the most free from putrefaction, can secure the anatomist from the danger of this formidable disease. Mr. Dease had been in the habit of dissection for more than twenty years. In Dr. Colles's recollection he found most of the instances of inflammation and fever following wounds received in dissection, among those pupils who had arrived at the third season of their anatomical pursuits. We are disposed to think Dr. Colles is not correct in supposing that it is a generally received opinion among the profession, "that the danger from wounds in dissection is in proportion to the putrefaction of the subject, or to the contagious nature of the disease which had caused death." The best informed anatomists of this country do not entertain such an opinion, their experience coinciding with that of our author as expressed in the following passage.

"On the contrary, according to my observation, unpleasant consequences have been so rare, where the subject was far advanced in putrefaction, as to induce me to think that putrefaction rather gives protection to the anatomist; nor could I trace any connexion between the disorder in question, and the contagious nature of the disease which had destroyed the subject of dissection, and surely during the winters of 1818-9, when fever raged so extensively in Dublin, we should have had some striking instances of such a connexion had it existed. Perhaps the majority of the affections in question have occurred in cases of examination of bodies to ascertain the cause of death, and where the body had not even been interred." 219.

Dr. Colles has never witnessed any of those dangerous constitutional affections which are mentioned by some authors, as attending the dissection of bodies in a state of putrefaction. In respect to the question whether we are to attribute these accidents to a poison or septic principle inoculated, or merely to the puncture itself, in a peculiar state of the constitution—we would be disposed to answer, in the absence of demonstrable proof, that there is great reason to suppose some poisonous matter introduced—the effects of this being modified by the state of the constitution at the time. The consequences of mere punctures by common instruments, in the various avocations of life, are very trivial, and, when they do become serious, are of a very different class from those following dissection wounds. We allude to the tetanic affections occasionally following slight wounds, especially in tropical climates.

As our author cannot offer any thing consolatory in the way of treatment, it is the more incumbent on anatomists, he reasonably thinks, to attend to the means of prevention.

"The advantages of the immediate application to such wounds of caustics in a solid or liquid form, is too obvious to require any comment; they are the only means upon which we can rely with absolute certainty.

"But those who are acquainted with the zeal of anatomists must doubt whether any practice which interferes with the prosecution of their pursuits will be generally adopted; the pain and the delay which must be occasioned by applying caustic to every little scratch, will prevent this plan from being as generally followed as it should be, or as the safety of individuals require.

"I would therefore recommend that each dissecting table be furnished with a cup of oleum terebinthinæ, into which the anatomist should plunge his finger the moment it is wounded. The smart which this produces is so inconsiderable, that it will not cause any serious delay, while the irritation may counteract the power of infection, or alter the mode of inflammation in the wound." 222.

We may be permitted to remark that, for many years past, the precept and example of Professor Chaussier have been adopted in the anatomical schools of Paris. Each student keeps in his pocket a small phial of the liquid butter of antimony (muriate of antimony) and whenever he wounds himself, in dissection, plunges the pointof a little wooden pencil into the caustic, and quickly cauterizes the puncture or wound. We, ourselves, would recommend the strong nitric acid, where the wound is tortuous, or made with a sharp pointed instrument, as this liquid immediately penetrates every part of the puncture, and completely disorganizes its parietes, thereby rendering them incapable of taking up any part of the septic principle. When, unfortunately, the poison has taken effect, we know of no *specific* means of checking its progress, and therefore we are obliged to combat the symptoms on general principles, for which no rule can be laid down. When we reflect that the inflammation produced by these punctures is of a specific kind, and attended with a peculiar constitutional derangement, we much doubt the propriety of that active system of depletion which is so effectual in common phlogosis and acute inflammatory diseases.

We shall review the other papers contained in this volume in our next number.

